# The Power of Faculty Development: The Impact on Teaching a Procedural Skill Framework

**DOI:** 10.7759/cureus.63279

**Published:** 2024-06-27

**Authors:** Katryna Thomas, Gayle Haischer-Rollo, Sabrina Silver, Jessica Servey, Diane Hale

**Affiliations:** 1 Surgery, Brooke Army Medical Center, San Antonio, USA; 2 Faculty Development, Uniformed Services University of the Health Sciences, Bethesda, USA; 3 Family Medicine, Indiana University Health Primary Care, Indianapolis, Indianapolis, USA; 4 Faculty Affairs, Uniformed Services University of the Health Sciences, Bethesda, USA

**Keywords:** sawyer framework, teaching procedures, graduate medical education, procedural skill framework, faculty development

## Abstract

Background: Teaching outpatient procedures is a skill often overlooked in faculty development. This oversight may lead to faculty employing a haphazard approach. Competency in procedural skills is inherent, and acquiring proficiency in procedural skills is necessary across all medical specialties, with some centers moving toward a blended simulation-based approach rather than the traditional Halstedian “see one, do one, teach one” mantra. While both formats have their pros and cons, they share the unifying concept of performance-based assessments and a standardized method for teaching procedures, which has typically been lacking a formal framework.

Objective: This study aimed to implement and evaluate the impact of teaching an educational technique in a multidisciplinary faculty education workshop about the Sawyer framework for psychomotor skill acquisition.

Methods: An interactive 90-minute workshop through the Uniformed Services University Faculty Development Program was developed and presented from February 2021 to October 2023 at multiple military treatment facilities. Participants enrolled in the workshop either by online registration or by walking in on the day of the workshop. A postworkshop survey was collected voluntarily. Through the survey, participants self-evaluated their current teaching strategy and made changes to their future strategy based on the framework they learned during the workshop. This was a mixed methods approach with quantitative survey data that were analyzed using Microsoft Excel (Microsoft Corporation, Redmond, WA) and qualitative data through thematic analysis using a constructivist inductive approach.

Results: There were 52 sessions with a total of 570 participants across 22 unique specialties. The response rate was 50%. Before the workshop, 22% of responding participants had no teaching strategy, and 49% had a partial but not explicit strategy for teaching. After the workshop, 89% of respondents answered that they would either implement a new or modify an existing strategy. Ninety-three percent of respondents reported that the Sawyer method was applicable to their future teaching. The overall themes from participants were that this procedural framework allowed for personal improvement in clear communication, individualized learner-centered teaching, and improved intentionality of teaching procedures.

Conclusion: Almost two-thirds of the faculty did not have a formal teaching method before this course, which is consistent with current data. Implementing a standardized framework for teaching procedures through faculty development workshops for multidisciplinary medical faculty educators can improve the educational quality of procedural skills.

## Introduction

Teaching outpatient procedures has become a core component of medical education. The Accreditation Council for Graduate Medical Education, American Board of Medical Specialties, and American Osteopathic Association have included it as one of the core competencies (patient care and procedural skills), and the American Association of Medical Schools and the American Association of Colleges of Osteopathic Medicine have listed it as one of their entrustable professional activities [1-5]. However, existing literature does not provide consensus on the optimal pedagogical strategy or assessment [6]. This gap is partly because teaching outpatient procedures is a skill often overlooked in faculty development. Only a handful of published material is found in this area, mostly focusing on single procedures [7,8]. A national survey of pediatric critical care faculty found that the ability to teach procedures was desired. However, almost 40% of respondents reported they had no formal teaching, and over half reported they would benefit from more instruction [9].

Current literature focuses on descriptive frameworks for structuring the procedural teaching experience, with little emphasis on practical steps for faculty skill acquisition [7-13]. Most of these frameworks blend simulation with the traditional Halstedian “see one, do one, teach one” mantra [14,15]. While both formats have pros and cons, there has been a lack of consistent formal frameworks with a concept of performance-based assessment for faculty to utilize for teaching procedures.

Numerous teaching theories underpin simulation and procedural education. Despite the increasing procedural aspects of healthcare, few described frameworks are specific to teaching procedures. Peyton’s four-step framework uses demonstration and increases the learner's active engagement in each step [16]. George and Doto described a similar five-step model that additionally considers the learner motivation and affective components of learning procedures [17]. Both frameworks are limited by a lack of describing the cognitive components of learning a procedure. The Sawyer six-step model is a framework that describes both the cognitive and technical aspects of procedural training. It is the only model that considers the maintenance of skill [18]. The Sawyer model was selected as the framework due to its applicability in competency-based procedural teaching in a broad variety of skills and specialties [19,20]. Sawyer’s model consists of the following steps [18].

1. Learn: The learner learns the conceptual basis of the procedure and observes it being performed.

2. See: The learner observes the procedure being performed step by step.

3. Practice: The learner practices the procedure on a simulator or with a partner.

4. Prove: The learner demonstrates the procedure to an evaluator.

5. Do: The learner performs the procedure on a patient under supervision.

6. Maintain: The learner maintains their skills through regular practice.

This study sought to investigate the impact of faculty attitudes and confidence toward teaching procedures after participation in a purposefully designed faculty development workshop. The workshop focused on imparting foundational skills for teaching procedures through a structured approach.

## Materials and methods

Uniformed Services University (USU) is a medical school within a large healthcare organization that uses 23 outlying teaching hospitals located across the United States. The USU Faculty Development Program provides foundational faculty development for these sites, which may have additional faculty development within specific departments. The program has a trained core set of faculty developers, starting in 2017, referred to as the Faculty Development Outreach and Certification (FOCUS) program [21]. This group delivers standardized faculty development for tangible skill improvement at their individual teaching hospitals for all specialties and other health professions (dentists, nurses, pharmacists, etc.). As part of the standardized program, the “Teaching Procedures” workshop was developed to provide practical and theoretical knowledge and reflection on procedural education. "Teaching Procedures" was a 90-minute workshop that took place from February 2021 to October 2023. Participants enrolled in the workshop via online registration or walking in on the day of the workshop and voluntarily participated in the optional postworkshop survey. During this specific workshop, the participants engaged with the Sawyer framework and physically simulated a procedure with one participant as the learner, one as the faculty, and one as the peer observer providing feedback. To create a generalizable procedure for the multispecialty learning environment, wrapping a small package (cell phone) was chosen as the neutral simulated procedure. There were also opportunities to engage with other learners to discuss current teaching practices and self-reflect on gaps in teaching procedures.

Data collection

**Figure 1:**
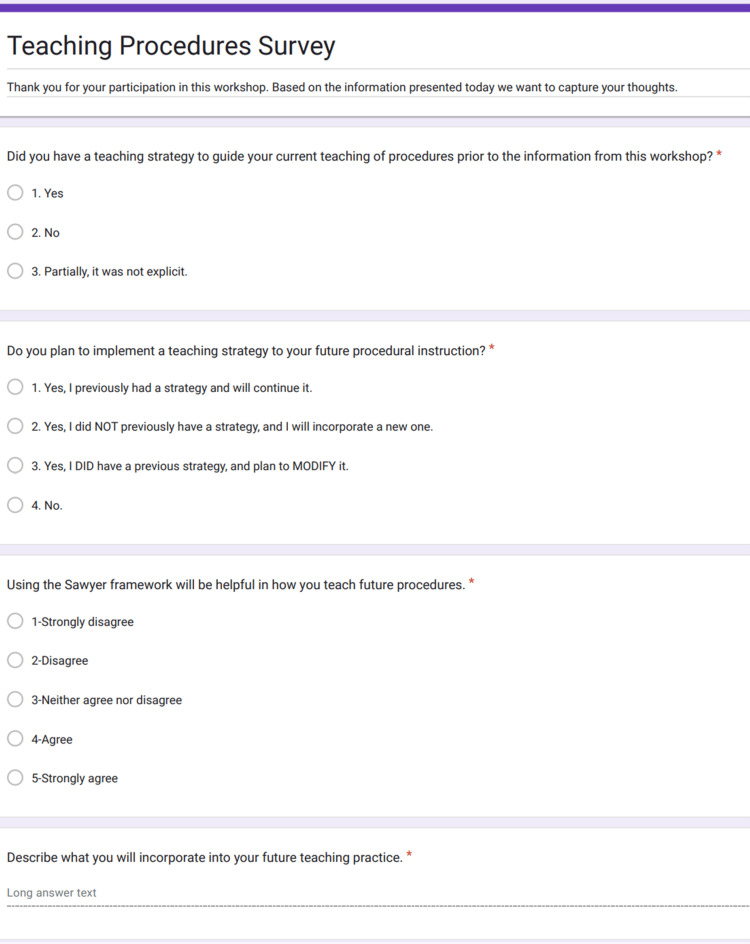
Questions accessed via QR code

The demographics data were self-reported through the USU faculty development online portal at the time of initial registration with the platform. Participants either registered in advance through the portal or attended the workshop as walk-ins. If this was their first USU faculty development workshop, they were sent instructions on how to create their online portal access postworkshop. A participant postworkshop survey was voluntarily collected to evaluate the impact and relevance to their educational strategies and daily practice. The questions developed by the workshop creators through USU FOCUS asked if the participants had a previous teaching strategy, if they plan to implement a strategy in the future, if they found the Sawyer framework helpful, and what aspects of the workshop they will include in their future teaching practice. Figure 1 has the complete wording of the questions and responses as the participant would see on their device. The local institutional review board has reviewed this curriculum, protocol DBS.2022.371, and has determined that it does not meet the criteria defined as research as part of a program evaluation.

Data analysis

This was a mixed-methods evaluation. The online survey was offered to participants for completion via a QR code (Figure 1) presented at the end of the didactic portion. Participants were encouraged to use their cell phones to complete the four-question survey at the workshop's end. The anonymized data were then exported into Microsoft Excel (Microsoft Corporation, Redmond, WA). The mode and percentage of the responses to each quantitative question were calculated in Excel. A chi-square test of independence was performed to examine the relationship between the participant's preexisting teaching framework and plan to implement a teaching strategy, and the plan to implement a teaching strategy and usefulness of the Sawyer framework using IBM SPSS Statistics for Windows, version 29 (IBM Corp., Armonk, NY). We used thematic analysis to analyze the open responses with a constructivist inductive approach [22]. Two of the authors (KT and SS) read through all the responses individually. One author (KT) performed the open coding and initial themes. Two authors (GH and JS) performed axial coding using the initial themes, and any disagreement was adjudicated through in-person discussion until a consensus was reached. Once that was completed, one author (SS) reviewed the final themes for consistency and reliability.

## Results

There were 52 sessions, with 570 attendees representing 22 unique specialties. Eighty-four surgical subspecialty attendees and 270 procedural specialty attendees attended the workshop (Table 1). A total of 288 responses were captured.

**Table 1:** Demographics of participants *Chiropractor, dietician, physician assistants, and social work ^a^Procedural specialties ^b^Surgical specialties

Participants	n (%)
College of Allied Health Sciences*	6 (1.1)
Graduate School of Nursing	12 (2.1)
Postgraduate Dental College^a^	75 (13.2)
School of Medicine	483 (84.7)
Anesthesiology^a^	33 (5.8)
Dermatology^a^	8 (1.4)
Family Medicine^a^	140 (24.6)
Gynecologic Surgery and Obstetrics^b^	34 (6.0)
Medical and Clinic Psychology	4 (0.7)
Internal Medicine	95 (16.7)
Military and Emergency Medicine^a^	20 (3.5)
Neurology	4 (0.7)
Pathology	8 (1.4)
Pediatrics	45 (7.9)
Pharmacology and Molecular Therapeutics	3 (0.5)
Physical Medicine and Rehab	9 (1.6)
Psychiatry	13 (2.3)
Radiology	9 (1.6)
Surgery^b^	50 (8.8)
No response	1 (0.2)
Total participants	570
Total workshop sessions	52

Quantitative responses

**Figure 2:**
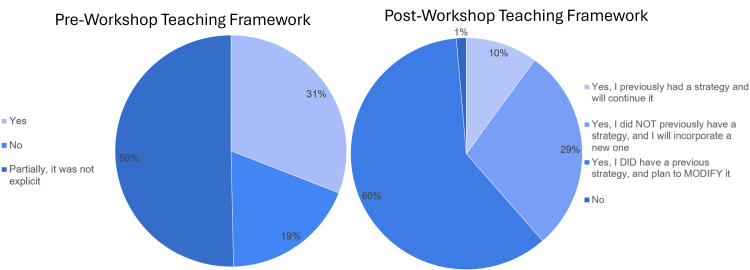
Pre- and postworkshop self-reflection on the framework for teaching procedures

**Figure 3:**
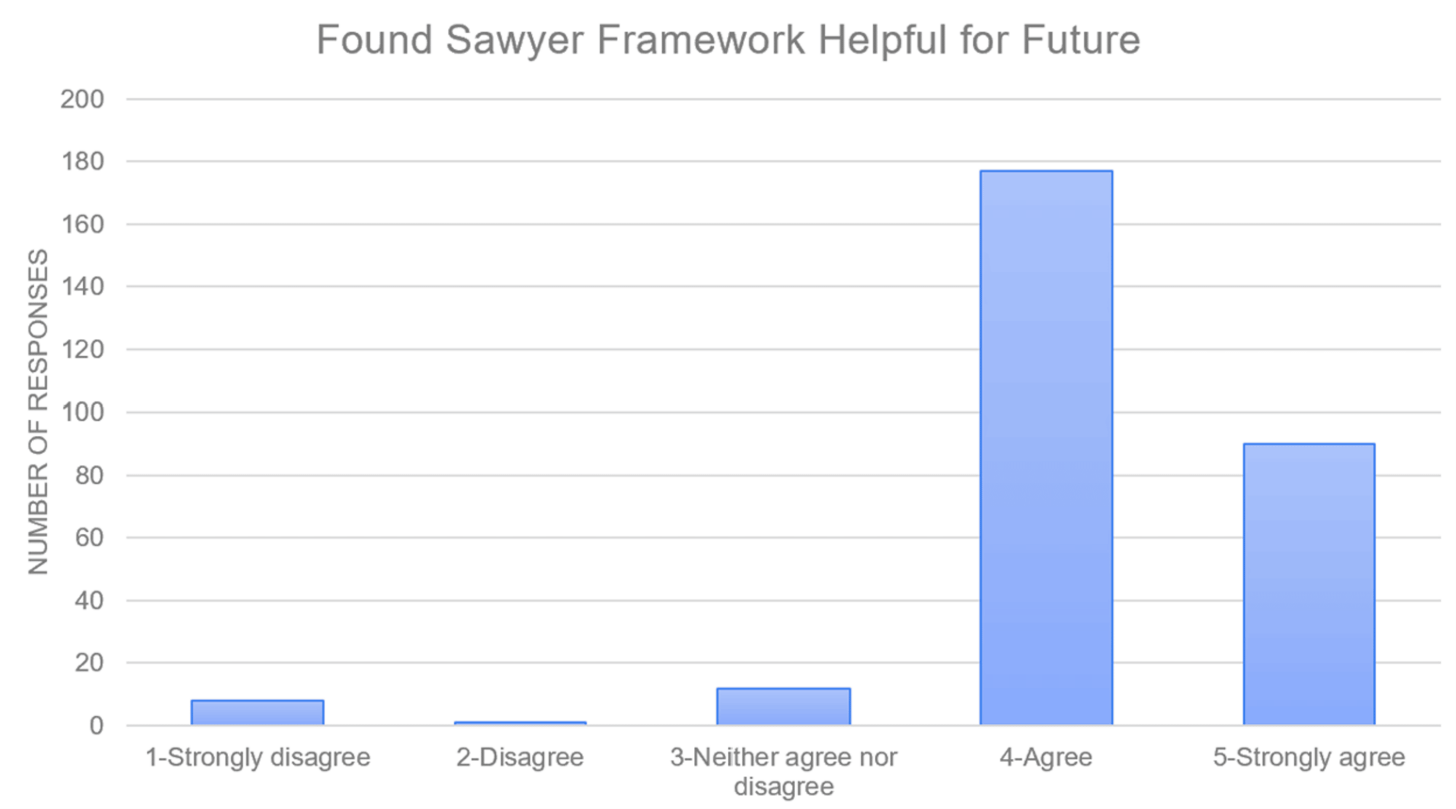
Participant response to future helpfulness of Sawyer framework

Approximately 50% of attendees responded to interactive surveys during the workshop. A total of 21.6% of participants reported no specific strategy for procedural teaching, 49% had a partial framework for teaching procedures, and 29% had a framework for teaching procedures (Figure 2). The participants were introduced to the Sawyer framework [18] during the workshop and interacted with the material through a simulated procedure experience using the framework skill they had learned. Upon completion of the workshop, 89% of respondents felt they would implement a new or modified strategy for teaching procedures, approximately 1% responded that they would not incorporate a teaching strategy, and 10% stated that they would continue using their previous framework (Figure 2). There was a statistically significant relation between attendees' previously established teaching framework and the plan to modify or incorporate a new framework (Table 2; p < 0.001). Ninety-three percent of participants felt that the Sawyer framework [18] would help shape their future procedural teaching, 3% felt it would not change their procedural teaching, and 4% were ambivalent (Figure 3). There was also a trend toward a significant relationship between the plan for implementing a teaching framework and the usefulness of the Sawyer framework for teaching procedures (Table 3; p = 0.06).

**Table 2:** Relationship between prior framework and plan for implementation of the future framework

X^2^ (6, N = 288) = 139.3, p < 0.001		Do you plan to implement a teaching strategy to your future procedural instruction?			
		Yes, I previously had a strategy and will continue it	Yes, I did NOT previously have a strategy, and I will incorporate a new one	Yes, I DID have a previous strategy, and plan to MODIFY it	No
Did you have a teaching strategy to guide your current teaching of procedures prior to the information from this workshop?	Yes	24	5	60	0
	No	2	43	6	3
	Partially, it was not explicit	3	34	107	1

**Table 3:** Relationship between plans for implementation of future framework and usefulness of Sawyer framework

X^2^ (12, N = 288) = 20.4, p = 0.06		Using the Sawyer framework will be helpful in how you teach future procedures				
		Strongly disagree	Disagree	Neither agree nor disagree	Agree	Strongly agree
Do you plan to implement a teaching strategy to your future procedural instruction?	Yes, I previously had a strategy and will continue it	3	0	4	17	5
	Yes, I did NOT previously have a strategy, and I will incorporate a new one	2	0	4	51	25
	Yes, I DID have a previous strategy, and plan to MODIFY it	3	1	4	105	60
	No	0	0	0	4	0

Qualitative responses

Of the 288 surveys collected, 273 (95%) participants answered the last prompt, which was an open-ended question geared toward identifying a future commitment to teaching practice. There were several themes, all of which pertained to being more deliberate in teaching procedures. The first theme was clear communication, which included subthemes of using precise language in explaining the steps of the procedure, communicating clear guidance, and recognizing that teaching communication is a part of the procedure in addition to the technical aspect. The second theme included individualized learner-centered teaching, which encompassed the subthemes of understanding the level of the learner, cocreating clear goals, maintaining clear expectations, and debriefing the procedure as part of feedback. The final theme was related to the intentionality of teaching procedures. In this theme, participants described subthemes of intentionally considering the cognitive part of procedures, the need for preparation before teaching, and being intentional about when to use simulation as part of the teaching strategy. While not themes, many comments mentioned teaching frameworks specifically reviewed during the session, such as Sawyer’s model and Robert’s briefing-intraoperative teaching-debriefing model [18,23]. Please refer to Table 4 for a full review of the themes, subthemes, and representative quotes from the responses.

**Table 4:** Participant themes on overall workshop takeaways AAR: after action review

Themes	Subthemes	Example comments
Clear communication	Precise language explaining steps, giving clear guidance, and communication as a part of the procedure	Focusing more on communication skills and not just technical skills and developing a method to evaluate skills over time
		I will try to use more precise language
		Improved communication and being explicit in my guidance
Individualized learner-centered teaching	Cocreation of goals, clear expectations, giving specific feedback, and recognizing the level of the learner (in the procedural skill)	Adaptive to students rather than “my-way” approach
		Working on assessing the learner's current knowledge of the procedure before launching into teaching the setup and process
		Being more deliberate in the AAR/feedback with goals for the learner's next attempt
		I want to be more direct before procedures, talking to the residents about their experiences and their goals for the procedure
Intentionality of faculty teaching	Awareness of the cognitive part of the procedure, adequate preparation before the procedure, consideration of when to use simulation, and use of checklists for assessment	I will try to be deliberate in supplying residents with prep materials to review, followed by a short video to watch before we do procedure clinic for the procedures scheduled that day
		I will then gauge their knowledge before the first actual procedure
		I will begin to be more intentional in the skills that I teach during each procedure, as well as focus on the counseling portion
		I am thinking of finding and using checklists for procedures since these can usually be anticipated and combined with global feedback

## Discussion

We examine whether a purposefully designed faculty development workshop focusing on foundational skills for teaching outpatient procedures through a structured approach will impact the attitudes and confidence of faculty participants. Our results align with the known literature that most faculty members are not given formal instruction on frameworks for teaching procedures despite most specialties requiring some form of procedural competency [7-9]. Our study demonstrates the lack of formal teaching among a variety of specialties in medicine. Through a standardized curriculum created for the USU Faculty Development program given by trained facilitators, this 90-minute workshop fills the gap of formal teaching for various specialties in medicine and other professions. One key to the FOCUS workshops is for faculty attendees to interact with the content, including deliberate built-in time for personal self-reflection. Another key to our workshops is fostering collegial interaction and sharing of best practices while increasing the awareness of challenges for all health profession educators. As such, it is notable that 13% of our attendees were from our Post-Graduate Dental College, who teach procedures to dentists in various dental specialties.

Our findings through a faculty development workshop demonstrated Sawyer’s six-step framework for teaching procedures, which is widely applicable to multiple specialties. Sawyer’s framework was cited in prior literature as a potential consideration for all health professions, especially utilizing simulation as part of an overall teaching pedagogy [24]. Our educational intervention demonstrated that the participants could add a teaching framework to their personal medical education toolkits. Our evaluation reinforces how faculty from many different health professions can learn tangible skills and experience attitudinal change for procedural teaching versus the traditional method of “see one, do one, teach one.”

Our program evaluation of this educational workshop results aligns with Kirkpatrick’s levels to evaluate training [25,26]. Kirkpatrick outcomes are described in four levels where level 1 is the learner’s reaction, level 2 is learning and can be divided into 2a (change in attitude) and 2b (change in skills), level 3 is behavior change, and level 4 is a change in the organization or patient care [25,26]. We assessed Kirkpatrick level 2 by asking faculty to predict potential changes in skills and acknowledge a change in attitude. The open-ended prompt provided insight into faculty explicitly describing potential future behavior change.

Our study has several limitations. While our study aims to assess the comfort level of procedural skill teaching, our data were collected using an “opt-in” survey after the course, which could lead to selection bias of participants, as only 50% completed the survey. Our study does, however, offer multidisciplinary perspectives through the heterogeneity of our participants. Another limitation is the self-reported bias that can occur after introducing the material; self-reported bias is typically lower in posttest experiences and, if present, underestimates the effect. Through our evaluation, we demonstrate an impact in a postexperience survey. Another limitation of this workshop is the limited ability to practice the teaching strategy only through simulation. The participants did not have the opportunity to practice the skills in the medical education setting after receiving feedback from a peer. Another unique aspect of this workshop is that it was presented by multiple different USU FOCUS facilitators from different specialties at different locations throughout the country. There is potential for minor variability in the teaching of material, but it is also a strength that this was easily reproducible with trained faculty through the USU FOCUS program. Our future directions could explore the internalization of the principles, and the next steps could include peer evaluation of the workshop participants in an in vivo teaching experience or a follow-up survey to the participants inquiring about their recent teaching experience and how/if they incorporated the Sawyer framework learned during their FOCUS workshop.

## Conclusions

A majority of graduate medical education faculty do not get formal training on teaching strategies for procedural education. Through the implementation of a multidisciplinary interprofessional faculty development session presenting and interacting with a dedicated procedural teaching framework, faculty can develop skills to structure their teaching. Scaffolded and intentional teaching practices can improve procedural teaching quality for learners.
